# Specific Cellular Incorporation of a Pyrene-Labelled Cholesterol: Lipoprotein-Mediated Delivery toward Ordered Intracellular Membranes

**DOI:** 10.1371/journal.pone.0121563

**Published:** 2015-04-15

**Authors:** Gérald Gaibelet, Sophie Allart, François Tercé, Vincent Azalbert, Justine Bertrand-Michel, Safouane Hamdi, Xavier Collet, Stéphane Orlowski

**Affiliations:** 1 INSERM U563/1048, CHU Purpan, 31024, Toulouse, cedex 3, France; 2 CEA, SB2SM and UMR8221/UMR9198 CNRS, I2BC, IBiTec-Saclay, 91191, Gif-sur-Yvette, cedex, France; 3 Université Toulouse III, UMR 1048, F-31000, Toulouse, France; 4 Plateau technique d’Imagerie Cellulaire, INSERM U1043, F-31300, Toulouse, France; 5 INSERM U1048, F-31400, Toulouse, France; 6 INSERM U1048, Lipidomic Platform Metatoul, F-31400, Toulouse, France; Oregon State University, UNITED STATES

## Abstract

In the aim of testing tools for tracing cell trafficking of exogenous cholesterol, two fluorescent derivatives of cholesterol, 22-nitrobenzoxadiazole-cholesterol (NBD-Chol) and 21-methylpyrenyl-cholesterol (Pyr-met-Chol), with distinctive chemico-physical characteristics, have been compared for their cell incorporation properties, using two cell models differently handling cholesterol, with two incorporation routes. In the Caco-2 cell model, the cholesterol probes were delivered in bile salt micelles, as a model of intestinal absorption. The two probes displayed contrasting behaviors for cell uptake characteristics, cell staining, and efflux kinetics. In particular, Pyr-met-Chol cell incorporation involved SR-BI, while that of NBD-Chol appeared purely passive. In the PC-3 cell model, which overexpresses lipoprotein receptors, the cholesterol probes were delivered via the serum components, as a model of systemic delivery. We showed that Pyr-met-Chol-labelled purified LDL or HDL were able to specifically deliver Pyr-met-Chol to the PC-3 cells, while NBD-Chol incorporation was independent of lipoproteins. Observations by fluorescence microscopy evidenced that, while NBD-Chol readily stained the cytosolic lipid droplets, Pyr-met-Chol labelling led to the intense staining of intracellular structures of membranous nature, in agreement with the absence of detectable esterification of Pyr-met-Chol. A 48 h incubation of PC-3 cells with either Pyr-met-Chol-labelled LDL or HDL gave same staining patterns, mainly colocalizing with Lamp1, caveolin-1 and CD63. These data indicated convergent trafficking downwards their respective receptors, LDL-R and SR-BI, toward the cholesterol-rich internal membrane compartments, late endosomes and multivesicular bodies. Interestingly, Pyr-met-Chol staining of these structures exhibited a high excimer fluorescence emission, revealing their ordered membrane environment, and indicating that Pyr-met-Chol behaves as a fair cholesterol tracer regarding its preferential incorporation into cholesterol-rich domains. We conclude that, while NBD-Chol is a valuable marker of cholesterol esterification, Pyr-met-Chol is a reliable new lipoprotein fluorescent marker which allows to probe specific intracellular trafficking of cholesterol-rich membranes.

## Introduction

Cholesterol is an essential component of mammalian cells, mainly present in the plasma membrane, but also distributed in various intracellular compartments. Indeed, it is both involved in membrane lipid composition, with a structural role and functional modulation of many membrane proteins, and in various cellular metabolic pathways and regulations. In order to maintain cellular homeostasis, cellular cholesterol content and trafficking are highly regulated at the level of influx, synthesis, esterification and efflux [1,2]. In the organism, cholesterol is provided both by synthesis in peripheral tissues and by absorption through the enterocytes in the intestine. Intestinal absorption requires solubilization in biliary micelles, which allows cholesterol availability in the aqueous medium present in the intestine lumen. In the other tissues, cellular exchanges of cholesterol with the external medium are mediated by lipoproteins, which act as “donor” and “acceptor” particles, ensuring a global regulation through its handling in the systemic circulation. The various lipoprotein classes display distinct physiological characteristics in connection with their specific receptors at the cell surface [3]. As a consequence, in some pathophysiological situations, the regulation of cholesterol metabolism and traffic is altered [4,5].

It is thus essential to be able to quantify and qualitatively characterize cellular cholesterol fluxes. Fluorescent markers present many technical and practical advantages over radiolabelled compounds [6], and they also allow imaging approaches [7]. However, very few fluorescent derivatives of cholesterol are presently available, and the question of the specificity of their cellular delivery is pivotal for assessing the physiological relevance of the cell staining obtained. Indeed, very few fluorescent cholesterol derivatives have been studied on cultured cells [8], and, when considering lipoprotein involvement, only high density lipoproteins (HDL)-mediated cell delivery of dehydroergosterol (DHE) [9] and of Bodipy-cholesterol (Bodipy-Chol) [10] have been tested. The use of the intrinsically fluorescent sterol DHE is however hampered by the drawback of a poor photostability (leading to quick fluorescence quenching), which presents heavy practical difficulties, and this is also the case for cholestatrienol and dansyl-cholesterol [8]. In contrast, the recently reported fluorescent derivative of cholesterol, Bodipy-Chol, appears much more convenient for biological labellings (cells and embryos, being possibly delivered by non-specific means, i.e. dimethylsulfoxide and cyclodextrin) [11], and it has been shown to insert into lipid rafts [12] and to be esterified in cells [11], making it a fair cholesterol probe for cellular metabolism. Few years ago, synthesis and chemico-physical characterization on model membranes of another new fluorescent cholesterol derivative, 21-methylpyrenyl-cholesterol (Pyr-met-Chol), has been reported, and it has been shown to interact with ordered membrane lipids similarly to cholesterol [13]. However, nothing is still known about its incorporation into cultured mammalian cells and its intracellular fate. In another hand, the other fluorescent cholesterol derivative, 22-nitrobenzoxadiazole-cholesterol (NBD-Chol), has been long ago reported to reliably probe cholesterol esterification [14], and in particular it has been repeatedly used for staining the cytosolic lipid droplets/bodies [15,16]. However, its pathway for cell incorporation has not been analyzed, and, otherwise, studies on model membranes have shown that it does not insert in ordered membranes as cholesterol [17,18]. In addition, we have recently shown that Pyr-met-Chol stably and specifically associates with lipoproteins, in clear variance with NBD-Chol [19]. Considering the contrasting chemico-physical properties of these two fluorescent cholesterol derivatives, and using the long ago described NBD-Chol as a “reference” probe, we thus wondered whether Pyr-met-Chol may be used as a reliable cholesterol probe in mammalian cultured cells regarding its trafficking, assessed by its intracellular distribution and/or metabolization downwards its lipoprotein-mediated cell delivery.

We have thus comparatively tested Pyr-met-Chol and NBD-Chol on two cultured cell models, both presenting high demands in cholesterol trafficking but largely differing by their general characteristics of lipid handling. We first used a cell model that incorporates exogenous cholesterol after its solubilization in mixed biliary micelles, which act as donors of lipids (and of the considered fluorescent derivatives of cholesterol), mimicking the intestine lumen conditions. For that purpose, we chose the Caco-2 cell line model, which displays an enterocytic differentiation allowing for studies of the intestinal absorption processes, including cholesterol uptake and transport [20]. Second, we used a cell model that specifically incorporates exogenous cholesterol thanks to lipoprotein receptors, i.e. scavenger receptor class B type I (SR-BI) for HDL and low density lipoprotein (LDL) receptor (LDL-R) for LDL, mimicking blood stream supplying. To test such a lipoprotein-specific vectorization of a fluorescent derivative of cholesterol, we chose the PC-3 cell line that originates from a metastatic, hormono-independent prostate cancer. PC-3 cells are known to require large amounts of cholesterol for growth and secretion of cholesterol-rich membrane vesicles, prostasomes, their increased cholesterol influx being ensured by an up-regulation of lipoprotein receptors [21]. This cell model thus provides an interesting pathophysiological situation, since cholesterol metabolism modulation could be considered as a potential approach for controling such a clinically devastating cancer [22]. In addition, it is noteworthy that the multifunction scavenger receptor, SR-BI, which is well-known as a HDL-specific receptor [23], has also been reported as a molecular receptor for biliary micelles [24,25].

In this work, we have successively addressed the specificity of the cell incorporation pathway of Pyr-met-Chol, as compared with NBD-Chol, and the characteristics of its intracellular distribution so obtained under fairly physiological conditions. In summary, we show here that (i) Pyr-met-Chol and NBD-Chol display clearcut differences for their cellular uptake processes, (ii) SR-BI is involved in Pyr-met-Chol, but not NBD-Chol, incorporation in Caco-2 cells from biliary micelles and in PC-3 cells from HDL, (iii) Pyr-met-Chol, stably associated with HDL and LDL in contrast to NBD-Chol, can be specifically delivered to PC-3 cells harboring the corresponding lipoprotein receptors, and (iv) Pyr-met-Chol incorporation leads to an intracellular staining of membrane compartments characterized by their cholesterol richness and their ordered structure, as revealed by pyrene excimer fluorescence emission. Finally, these data lead to the conclusion that, while NBD-Chol is a fair marker of esterification metabolism, Pyr-met-Chol is a reliable tracer of unesterified cholesterol of exogenous origin.

## Experimental Procedures

### Chemicals and cells

Pyr-met-Chol was a kind gift from Dr A Lopez (CNRS Toulouse, France) and stock solution was solubilized in chloroform/methanol (9:1), NBD-Chol was from Sigma-Aldrich and stock solution was solubilized in chloroform/methanol (2:1). Taurocholate (TC), Nile Red, filipin and recombinant albumin were from Sigma-Aldrich, Bodipy493 was from Molecular Probes, TMP-153 was from Biomol International LP, oleic acid, 1-monooleoylglycerol, phosphatidylcholine, lysophosphatidylcholine and 25-hydroxycholesterol were from Avanti, BLT-1 (2-hexyl-1-cyclopentanone thiosemicarbazone) was from Chembridge. Monoclonal mouse antibodies (Ab) against early endosome antigen-1 (EEA-1), against lysosomal-associated membrane protein-1 (Lamp-1), against CD13 and against CD63 were from BD Biosciences, monoclonal mouse Ab against calnexin was from Interchim, polyclonal rabbit Ab against adipocyte differentiation-related protein (ADRP)/adipophilin and against caveolin-1 were from Santa-Cruz, goat Alexa546-labelled anti-rabbit secondary Ab was from Invitrogen, donkey Cy3-labelled anti-mouse secondary Ab was from Jackson Immunoresearch Labs.

The Caco-2 cell line, clone TC7, was a kind gift from Dr M. Rousset [26]. It is originated from a differentiated human colon adenocarcinoma. Cells were cultured in DMEM medium supplemented with 20% heat-inactivated fetal bovine serum, 1% non-essential amino acids and 1% antibiotics (Invitrogen) in an incubator at 37°C in 10% CO_2_ and humidified atmosphere. They were seeded either on a solid (Nunc) or a porous support (Transwell, 0.4 μm pores polycarbonate filter). Culture medium was changed every 2 days. Cultured cells spontaneously form an epithelial-like polarized cell monolayer that is considered as well differentiated (as regarding functional enzymes and transporters expression) beyond 2 weeks after confluency [27].

The PC-3 cell line is originated from a human androgen-independent prostate cancer [28]. Cells were provided from ATCC. They were cultured in Ham’s F12 medium supplemented with 10% fetal calf serum and penicillin (100 U/ml) plus streptomycin (10 μg/ml) (Invitrogen) in an incubator at 37°C in 5% CO_2_ and humidified atmosphere, after seeding onto glass slides for microscopy observations or onto plastic supports for kinetics determinations. The plastic microplates suited for UV measurements were from Greiner, and those for visible measurements were from Nunc.

Stable expression of fluorescently tagged SR-BI in Caco-2 cells and PC-3 cells was achieved using a previously described lentivirus-based strategy, which permits efficient and long-term transgene expression without clone selection [29]. Briefly, fragments corresponding to the SR-BI open reading frame lacking the initiation codon were inserted downstream the enhanced green fluorescent protein (EGFP) cDNAs. The fusion fragments EGFP-SRBI were cloned into a HIV-1-based lentiviral pTrip vector carrying a tetracycline response element (TRE) (BIVIC platform, IFR 150, CHU Rangueil, Toulouse), in order to induce EGFP-SRBI expression after addition of 1 μg/ml doxycycline in the culture medium. The engeneered, EGFP-SR-BI-expressing Caco-2/EGFP-SR-BI and PC-3/EGFP-SR-BI cells were cultured under the same conditions as Caco-2 and PC-3 cells, respectively.

### Fluorescent labelling of purified lipoproteins

Purified LDL and HDL fractions were prepared from healthy donor plasma by ultracentrifugation (120 000 g, at 4°C, 66 h) onto a KBr layer of density 1.06 and 1.19, respectively. Fluorescent labelling was performed as previously described [19]. Briefly, 1–7 mg/ml of lipoprotein, dialyzed against phosphate-buffered saline (PBS), was incubated with Pyr-met-Chol (using ethanol as a vehicle) at 5% mol/mol of the non-esterified cholesterol amount present in the lipoprotein fraction (corresponding to 20–50 μM for HDL and 130–300 μM for LDL). Incubation was performed for 48 h at 37°C, under gentle stirring and protection from light, and labelled lipoproteins were then dialyzed using a semi-permeable membrane with a 10 kDa cutoff. Bound Pyr-met-Chol fluorescence was measured by a fluorometric assay, using a Varioskan Flash fluorescence plate reader (Thermo-Electron), with excitation and emission wavelengths at 330 nm and 400 nm respectively, against a standard curve. Protein concentration was determined by the Biorad protein assay, taking albumin as a standard.

### Analysis of cholesterol derivatives esterification

Acyl-CoA:cholesterol acyltransferase (ACAT) activity was determined on PC-3 cells cultured for 72 h in the presence of either Pyr-met-Chol (10 μM) or NBD-Chol (10 μM), then washed twice with PBS containing taurocholate (TC) 2 mM and once with PBS alone. Cells were then scrapped in methanol/water 2:1 v/v, and lipids extracted, separated on silica gel plate in hexane/formic acid/diethylether (55:1:45 v/v/v) and revealed by UV irradiation or iodide vapor.

### Cell incorporation and efflux kinetics measurement

Caco-2 cells were seeded at 300,000 per well in 12-well plastic plates. Between 15 and 21 days after cell confluency, cells were cultured overnight without serum, then washed and incubated during various periods with a bile salt micellar solution composed of 2 or 5 mM taurocholate, 0.6 mM oleic acid, 0.3 mM 1-monooleoylglycerol, 0.04 mM phosphatidylcholine, 0.16 mM lysophosphatidylcholine and 5 μM of Pyr-met-Chol or NBD-Chol.

PC-3 cells were seeded at 250,000 per well in 12-well plastic plates, and cultured until confluency, washed and then incubated with 5 μM of both Pyr-met-Chol and NBD-Chol using ethanol as a vehicle (final volume 0.5% v/v in the culture medium), under various culture conditions. For some experiments, incubations was performed with 0.1 mg/ml Pyr-met-Chol-labelled purified LDL or HDL (Pyr-met-Chol added at 5% mol/mol of the non-esterified cholesterol amount in the lipoprotein fraction) in the absence of fetal calf serum.

For incorporation experiments, after various incubation durations, Caco-2 or PC-3 cells were washed twice with PBS/TC 2 mM and once with PBS alone, and they were then lysed by sodium dodecylsulfate (SDS) 0.5% in PBS. Fluorescence of the cell lysate was quantified (for Pyr-met-Chol, excitation and emission at 330 nm and 400 nm, respectively; for NBD-Chol, 470 nm and 530 nm respectively) against standard curves realized under the same conditions (SDS 0.5% and the presence of control cell extracts). Each quantitative determination of cellular incorporation is normalized to the protein content of the cells. Data are representative of 2 to 9 independent experiments, each experimental point being performed in triplicate. After a 48 h standard PC-3 cell incubation, about 30–50% of Pyr-met-Chol was incorporated into the cells in absence of serum, compared to about 5–10% in the presence of serum, about 5% in presence of labelled purified HDL and about 50% in the presence of labelled purified LDL.

For basal efflux kinetics measurements, Caco-2 cells were first seeded on Transwell inserts (at 300,000 per well), and between 15 and 21 days after cell confluency, cells were incubated, as for the incorporation experiments, with a taurocholate-based micellar solution composed of 2 mM taurocholate and containing 5 μM of both Pyr-met-Chol and NBD-Chol, but added into the apical culture medium. After various incubation durations, cells were washed twice with PBS/TC 2 mM and once with PBS alone, and they were then lysed by SDS 0.5% in PBS, and fluorescence of the cell lysate was quantified as described above. In parallel, aliquots were taken from the basal culture medium, and fluorescence was quantified. For efflux kinetics measurements, PC-3 cells were incubated with Pyr-met-Chol as for the incorporation experiments during 48 h, then washed twice with PBS/TC 2 mM and once with PBS alone, and then cells were further cultured in the absence of Pyr-met-Chol for various periods, then washed, lysed and collected, and their remaining content in Pyr-met-Chol was determined as described above.

When appropriate, statistical significance of differences measured between two experimental conditions were assessed according to Student’s t test (p = 5% being taken as the significance threshold).

### Cell staining and immunofluorescence

Caco-2 cells were seeded either onto glass slides put in the same plastic wells as for cellular kinetics experiments, or on Transwell inserts whose filters were cut out and transfered onto glass slides for microscopy observation. Between 15 and 21 days after cell confluency, cells were cultured overnight without serum, then washed and incubated, as described above, with a taurocholate-based micellar solution containing 5 μM of both Pyr-met-Chol and NBD-Chol (added into the apical culture medium for cells cultured on filters). PC-3 cells were seeded onto glass slides put in the same plastic wells as for cellular kinetics experiments, and Pyr-met-Chol and NBD-Chol were added to the medium as described above (either directly, using ethanol as a vehicle, or by the mean of labelled purified lipoproteins), for 48–72 h, under various culture conditions. Caco-2 or PC-3 cells were washed twice with PBS/TC 2 mM and once with PBS alone, and fixed by paraformaldehyde (3%) during 12 min at 4°C. For staining of polar lipids in membranes, the fixed cells were treated by 1 μg/ml Nile Red, and for neutral lipids (in lipid droplets), they were treated by 1 μg/ml Bodipy493 during 15 min at room temperature (RT). For free cholesterol staining, cells were first fixed by paraformaldehyde (3%) during 30 min at RT, then treated by glycine 20 mM during 10 min (RT), and then by filipin at 70 μM during 30 min (RT). For immunofluorescence experiments, fixed cells were permeabilized by either Triton X100 0.01% during 5 min (RT) or saponin 0.05% during 45 min (RT), and are then blocked by bovine serum albumin 1 mg/ml or fetal calf serum 10%. The cells were incubated in the presence of primary Ab during 1 h at RT after Triton X100 treatment, or during the 45 min of saponin treatment, then in the presence of the secondary Ab during ½ h at RT. Each staining step was followed by three washes using PBS. The slides were finally mounted using Mowiol (Sigma). For live cell imaging, cells were maintained at 37°C and 5% CO_2_ within a small incubation chamber where humidity was regulated by a culture medium infusion system (Harvard Apparatus, France), during time lapse experiments in two-photon microscopy. Images were acquired at various times during 24 h.

### Mono-photon and two-photon excitation fluorescence microscopy imaging

Pyr-met-Chol and NBD-Chol were observed using a two-photon excitation (TPE) LSM 7MP Zeiss (Carl Zeiss, Jena, Germany) microscope equipped with a Chameleon Ultra II Ti:sappire laser (Coherent, Santa Clara, CA, USA), or a Leica SP5 (Leica Microsystems CMS, Mannheim, Germany) microscope equipped with a MAITAI Ti:sappire laser (Spectraphysics, Santa Clara, CA, USA). Immunofluorescence observations of Cy3 or Alexa-546 coupled secondary Ab staining were performed with the Leica SP5 microscope using the mono-photon excitation (MPE) confocal mode with a He:Ne laser 543 nm, allowing parallel observations on three channels tuned for precise wavelengths, and sequential use of TPE or MPE mode. For some experiments, we took advantage of the well-known spectroscopic property of the pyrene fluorophore to emit in the 420–560 nm range when an excited molecule transiently encounters a non-excited molecule, making an “excited dimer” or excimer. In particular, this happens when Pyr-met-Chol is inserted in a cholesterol-rich ordered membrane domain [30,31]. Thus, Pyr-met-Chol was excited at a wavelength of 710 nm in TPE, and we used either i) a 370 to 485 nm band-pass (BP) filter to select emission wavelengths of Pyr-met-Chol global fluorescence emission, ii) or a 370 to 409 nm BP filter for Pyr-met-Chol monomer emission, iii) or a 443 to 507 nm BP filter for excimer emission. For NBD-Chol and Bodipy493, excitation was at 940 nm in TPE, and emission recorded with a 500–550 nm BP filter. For Nile Red, excitation was at 810 nm in TPE, and emission recorded with a 530–640 nm BP filter; for filipin, wavelengths were 710 nm and 370/485 nm respectively; for EGFP, wavelengths were 940 nm and 490/540 nm respectively. Excitation of Cy3 or Alexa-546 coupled secondary Ab staining were performed in MPE with the laser line 546 nm, and emission was selected with a 565–610 nm BP filter. Acquisitions were performed with a 63X oil objective (1.4 NA). Fields were averaged 4 times to increase signal to noise ratio, and amplifier gain and offsets of each photomultiplier were adjusted for a given experiment, but were set constant for the sake of comparison between two coupled conditions, except otherwise indicated. Each condition was imaged on at least three different fields.

Image processing and analysis was performed using Metamorph Imaging System software package (Molecular Devices Corp., Sunnyvale, CA). At least 20 cells were quantified for each staining. Colocalization analyses for Pyr-met-Chol were done with the JACoP plugin of Image j software [32]. Manders M2 overlap coefficient was calculated for each couple, defined as the ratio of the summed intensities of pixels from the Pyr-met-Chol channel for which the intensities of the red channel was above zero to the total intensity in the Pyr-met-Chol channel [33]. Therefore, M2 is a good indicator of the proportion of the Pyr-met-Chol signal coincident with a signal in a red channel over its total intensity.

## Results

### Comparison of the cellular behaviors for NBD-Chol and Pyr-met-Chol incorporated in Caco-2 cells

**Fig 1:**
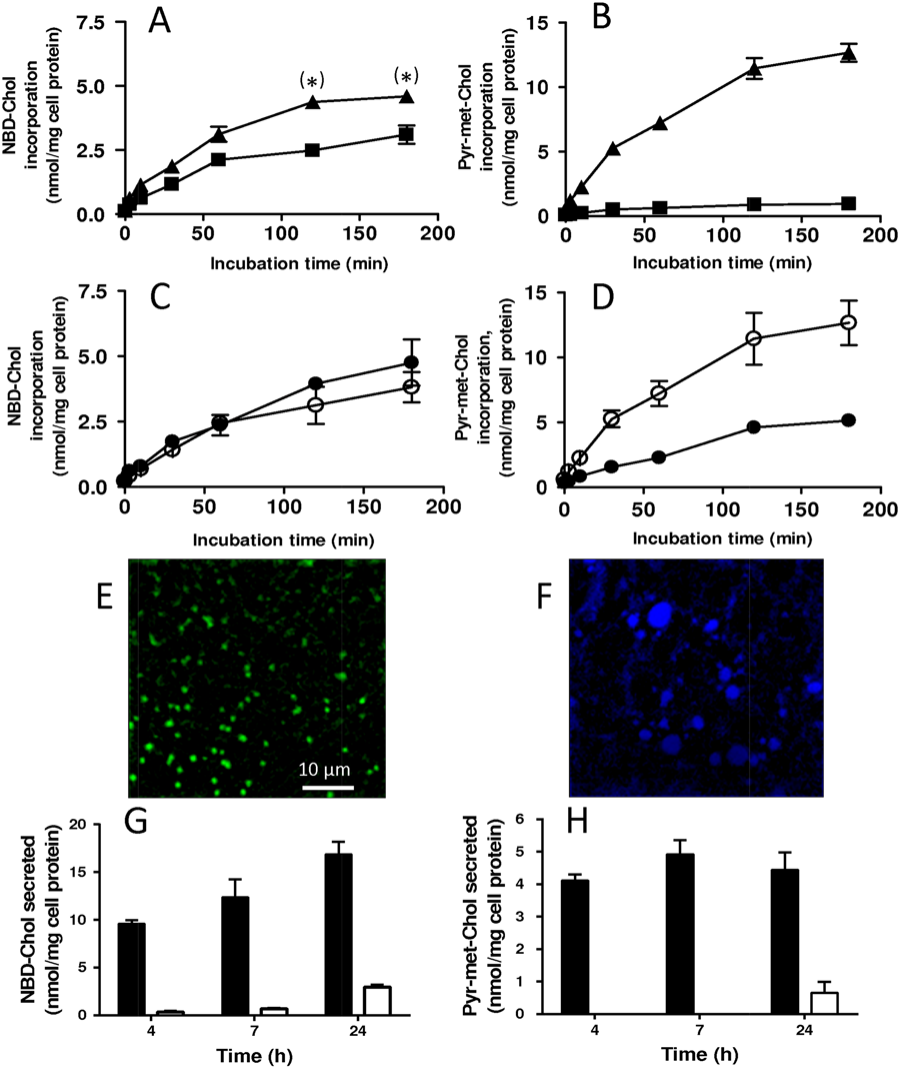
Compared cellular incorporations and effluxes of NBD-Chol and Pyr-met-Chol in Caco-2 cells. *Panels A and B*: Influence of the concentration of the solubilizing bile salt taurocholate on cellular incorporation. Differentiated Caco-2 cells cultured on solid support were incubated for various periods with a solution composed of 2 (squares) or 5 mM (triangles) taurocholate, 0.6 mM oleic acid, 0.3 mM 1-monooleoylglycerol, 0.04 mM phosphatidylcholine, 0.16 mM lyso-phosphatidylcholine, and 5 μM of NBD-Chol (panel A) or Pyr-met-Chol (panel B). Cellular contents of NBD-Chol and Pyr-met-Chol were quantified by fluorometry on cells lysed in the presence of 0.5% SDS. When non-apparent, error bars are included within the symbols; p<5% (*) indicates a statistically significant difference between the absence and presence of taurocholate in Panel A; in Panel B, the difference is highly significant between the whole kinetic curves. *Panels C and D*: Influence of the SR-BI inhibitor BLT-1 on cellular incorporation. Differentiated Caco-2 cells cultured on solid support were incubated for various periods with the same solution than in Panels A and B, containing 5 mM taurocholate with 5 μM of Pyr-met-Chol or NBD-Chol, in the absence (hollow circles) or presence (closed circles) of 10 μM BLT-1. Cellular contents of NBD-Chol and Pyr-met-Chol were quantified by fluorometry on cells lysed in the presence of 0.5% SDS. When non-apparent, error bars are included within the symbols; in Panel D, the difference is highly significant between the whole kinetic curves. *Panels E and F*: Cell staining. Differentiated Caco-2 cells cultured on porous support were incubated for 2 hours with the same micellar solution than in Panels A and B, containing 5 mM taurocholate with 5 μM of Pyr-met-Chol or NBD-Chol, added in the apical compartment, then fixed and observed by TPE fluorescence microscopy; NBD channel is green (panel E) and pyrene channel is in blue (panel F). Scale bar corresponds to 10 μm. *Panels G and H*: Compared cells incorporation and efflux kinetics. Differentiated Caco-2 cells cultured on porous support were incubated for various periods with the same solution than in Panels A and B, containing 2 mM taurocholate with 5 μM of Pyr-met-Chol or NBD-Chol, added in the apical compartment. Cellular (dark bars) and basal compartment (white bars) contents of NBD-Chol (panel G) and Pyr-met-Chol (panel H, note the different ordonate scale) were quantified by fluorometry on cells lysed in the presence of 0.5% SDS.

We first addressed the cell incorporation of the two fluorescent derivatives of cholesterol by measuring their respective uptake kinetics into Caco-2 cells, cultured on solid support and incubated for various durations with taurocholate-based mixed micelles containing 5 μM of NBD-Chol or Pyr-met-Chol. When taurocholate concentration increased from 2 mM, close to its critical micellar concentration (cmc), to 5 mM, clearly above it, NBD-Chol incorporation was only slightly increased (for 2 and 3 h incubations, with an increasing factor of 1.4 at 3 h), whereas Pyr-met-Chol incorporation increased more than 10 times after a 3 h incubation (Fig 1A and 1B). Using 5 mM taurocholate to form the donor micelles, we then tested the effect of the presence during the incubation of BLT-1 (at 10 μM), an inhibitor of SR-BI-mediated lipid apical uptake by enterocytes [34]. While NBD-Chol uptake was not altered by BLT-1, Pyr-met-Chol uptake was reduced by more than 60% (Fig 1C and 1D).

We then performed imaging studies using confocal fluorescence microscopy to compare the cell staining patterns obtained with NBD-Chol and Pyr-met-Chol. Due to the low excitation wavelength of pyrene, we used TPE microscopy. Control images of non-stained cells showed an absence of detectable autofluorescence. We tested the respective cellular localizations of the probes in Caco-2 cells cultured on porous support since this is the most physiological situation, with highly differentiated and polarized cells forming a sealed cellular monolayer. After incubation with the taurocholate-based donor micelles added into the apical compartment, co-staining of the Caco-2 cells by the two fluorescent derivatives of cholesterol clearly showed that they did not stain the same intracellular structures (Fig 1E and 1F; see the merge image in panel A in S1 Fig). The rather punctate structures intensely stained by NBD-Chol could be assigned to lipid droplets/bodies, since they exhibited a good colocalization with ADRP/adipophilin (panel B in S1 Fig). Actually, NBD-Chol has been reported on various cell systems to be esterified by ACAT (acyl-CoA:cholesterol acyltransferase), even more efficiently than cholesterol, and embedded into lipid bodies, hence leading to a huge fluorescence emission enhancement due to the hydrophobic environment of the NBD fluorophore in the core of these lipid bodies [14,15,16].

We also measured the respective cellular efflux kinetics of NBD-Chol and Pyr-met-Chol to the basal compartment from Caco-2 cells cultured on porous filters and submitted to an apical incubation with the taurocholate-based donor micelles. Since we considered longer incubation durations, up to 24 hours, than for the uptake experiments shown in Fig 1A and 1B, we used the lower taurocholate concentration of 2 mM to avoid cell suffering. We simultaneously measured the corresponding cellular incorporation of the two probes, which showed that the amount of NBD-Chol incorporated still increased over 24 h, whereas that of Pyr-met-Chol was stationary between 4 and 24 h (Fig 1G and 1H). The amount of NBD-Chol effluxed in the basal compartment progressively increased between 4 and 24 h, whereas that of Pyr-met-Chol was much limited and only detectable after the 24 h incubation (Fig 1G and 1H).

These experiments on Caco-2 cells indicated marked differences for the cellular behaviors of the two fluorescent cholesterol derivatives: Pyr-met-Chol absorption involved SR-BI, whereas NBD-Chol is able to incorporate the cultured cells by a purely passive diffusion mechanism (likely thanks to its amphiphilic nature).

### Pyr-met-Chol delivery to PC-3 cells via lipoprotein-specific uptake pathways

**Fig 2:**
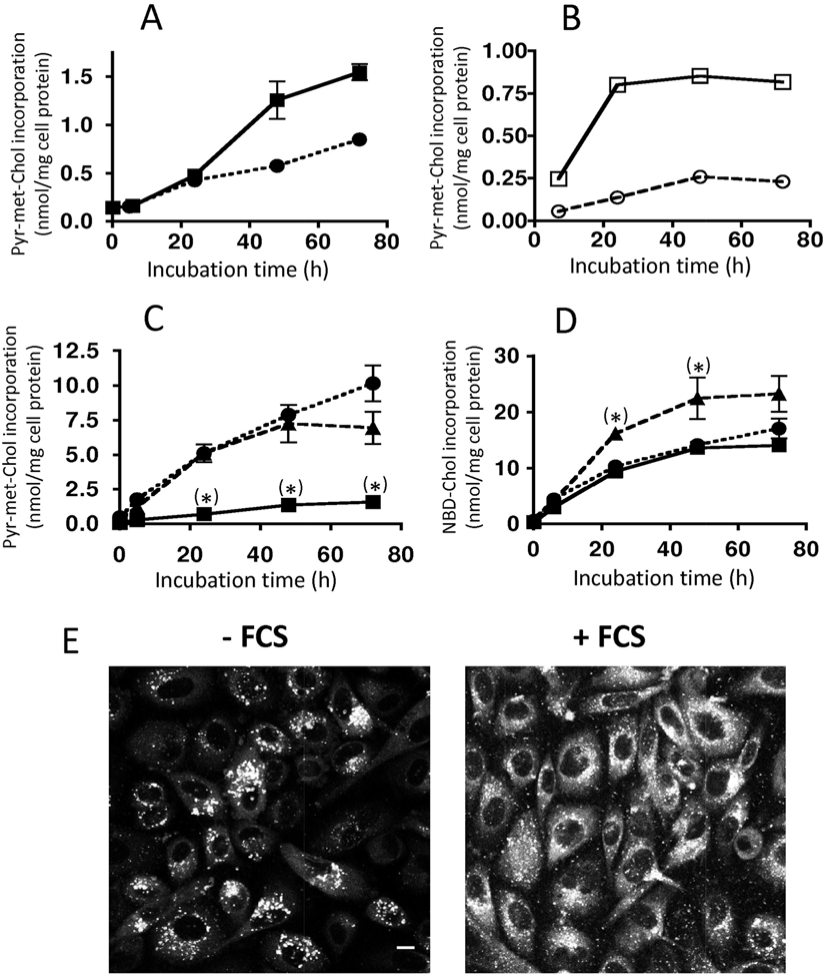
Donor-dependence of the incorporation kinetics of Pyr-met-Chol and NBD-Chol in PC-3 cells. *Panels A and B*: PC-3 cells were incubated for various periods with 5 μM Pyr-met-Chol in Ham F12 medium supplemented with 10% fetal calf serum (closed squares) or 10% of human serum (closed circles), or with 0.1 mg/ml of Pyr-met-Chol-labelled purified LDL (open squares) or Pyr-met-Chol-labelled purified HDL (open circles). In Panel B, the difference is highly significant between the whole kinetic curves. *Panels C and D*: PC-3 cells were incubated for various periods with 5 μM Pyr-met-Chol (panel C) or NBD-Chol (panel D) in Ham F12 medium in the absence (triangles) or in the presence of 10% fetal calf serum (squares) or 1 mg/ml human recombinant albumin (circles). Pyr-met-Chol and NBD-Chol contents were measured by fluorometry on cells lysed in the presence of 0.5% SDS. When non-apparent, error bars are included within the symbols; p<5% (*) indicates a statistically significant difference between the absence and presence of serum (or albumin). *Panel E*: PC-3 cells were incubated for 24 h with 5 μM Pyr-met-Chol in the absence (left image) or presence (right image) of 10% fetal calf serum, and observed by TPE fluorescence microscopy as living, non-fixed cells. In order to present images with adequate signal intensities giving them a fair legibility, we used a higher acquisition gain for the image recorded in the presence of serum with respect to that in the absence of serum. Scale bar corresponds to 10 μm.

Taking into account that NBD-Chol only marginally associates with HDL and not detectably with LDL, while Pyr-met-Chol associates with serum lipoproteins grossly proportionally to their protein content [19], we focussed on Pyr-met-Chol-labelled lipoproteins as donor particles to target cells. We thus measured the incorporation of Pyr-met-Chol in PC-3 cells, known to display up-regulated LDL receptors [21,35]. Pyr-met-Chol was either added directly to the serum-containing culture medium or delivered from labelled purified lipoproteins. When Pyr-met-Chol (5 μM) was incubated with PC-3 cells in the presence of fetal calf serum or human serum (at 10%), there was a comparable increasing uptake over 72 h (Fig 2A), even with a tendency for a higher incorporation for fetal calf serum, but likely without physiological significance taking into account the comparable serum composition, in particular regarding cholesterol [36]. At variance, when Pyr-met-Chol was previously associated with purified lipoproteins, the uptake was saturable with LDL, with a clearly more rapid incorporation and with a higher plateau level (3.5 times) for LDL than the level reached at 72 h for HDL (Fig 2B). Taking into account a labelling level by Pyr-met-Chol higher for HDL than for LDL by a factor of about 2 (when normalized against protein content) [19], this difference of cell incorporation is actually twice more marked between LDL and HDL. This clear difference between the kinetics of cell incorporation according to the nature of the lipoprotein delivering Pyr-met-Chol suggested that it can follow different lipoprotein-specific uptake pathways. In addition, as a control experiment, when Pyr-met-Chol was incubated with PC-3 cells in the absence of serum or in the presence of recombinant albumin (devoid of any lipoprotein contaminant) instead serum, the cellular incorporation was much more important (Fig 2C). This was consistent with a chelating effect of the lipoproteins for Pyr-met-Chol that prevents it to freely diffuse in the medium and to be non-specifically incorporated into the cells. Since the kinetics in the presence of albumin was not different from that in the absence of serum, albumin was thus not involved in the specific cellular uptake pathway, specifically mediated by LDL or HDL. In contrast, the cellular uptake of NBD-Chol, virtually not associated with lipoproteins, only moderately (but significantly, p<5% for 24–48 h incubation times) depended on the presence of serum in the incubation medium, and albumin presence was sufficient to mimic the effect of serum on this non-specific cellular uptake (Fig 2D). In addition, fluorescence microscopy imaging did not show any difference whether NBD-Chol was incorporated in the absence or presence of serum (not shown).

Furthermore, when PC-3 cells were incubated for 48 h in the presence of 10 μM 25-hydroxycholesterol, a liver X receptor (LXR) agonist regulating cell cholesterol metabolism and traffic, cellular incorporation of Pyr-met-Chol was decreased by about 25% (p<5%) (panel A in S2 Fig), while that of NBD-Chol was not significantly altered (decrease <10%) (panel B in S2 Fig). This differential effect provided further evidence that Pyr-met-Chol, in contrast to NBD-Chol, was incorporated through a regulated specific pathway. In addition, the presence of BLT-1 (at 10 μM), a SR-BI-mediated HDL uptake inhibitor [37], during the PC-3 cells 48 h incubation with purified Pyr-met-Chol-labelled HDL or LDL induced a decrease of Pyr-met-Chol incorporation by about 50%, compared to the control without BLT-1, in the case of HDL (p<5%) (panel C in S2 Fig), but had no effect in the case of LDL (panel D in S2 Fig): this showed the selective involvement of SR-BI for the HDL-mediated incorporation. By the way, the absence of inhibiting effect of BLT-1 when using labelled purified LDL is indicative that Pyr-met-Chol labelling does not modify LDL in such a manner to be handled by SR-BI instead of their specific receptors (like oxidized or acetylated LDL). Finally, the amount of Pyr-met-Chol specifically incorporated into the PC-3 cells, about 1 nmol/mg cell prot, was rather low but nevertheless represented a non-negligeable pool compared to their cellular cholesterol, measured to be 27 ± 3 nmol/mg cell prot for non-esterified cholesterol and 3 ± 0.3 nmol/mg cell prot for esterified cholesterol.

The role of serum components in the cellular Pyr-met-Chol incorporation was further analyzed by fluorescence microscopy imaging of the intracellular staining of living PC-3 cells, incubated 24 h in the presence or absence of fetal calf serum. In the presence of serum, we observed that Pyr-met-Chol fluorescence was distributed between intracellular punctate structures and a diffuse cytoplasmic staining (Fig 2E), with an intensity ratio of 0.21 for the brightest structures to the total cellular fluorescence emission. In clear contrast, in the absence of serum as control conditions, Pyr-met-Chol fluorescence was predominent in the punctate structures with respect to a much lower diffuse cytoplasmic staining (Fig 2E), with an intensity ratio of 0.43. This difference of intracellular staining patterns showed that the corresponding cell incorporation pathways were distinct, and this strongly supported that, in the presence of serum, Pyr-met-Chol did not incorporate the cells as free molecules in the medium, as if they were released by the labelled lipoproteins (hence bypassing their receptors as in the absence of serum). Globally, it can be concluded that the serum lipoproteins mediate Pyr-met-Chol cellular incorporation by specific delivery routes, giving the opportunity to address the differences of cell trafficking downstream HDL and LDL receptors. In contrast, NBD-Chol incorporation in PC-3 cells, as in Caco-2 cells, appears independent of specific receptors.

### Intense staining of intracellular punctate structures by Pyr-met-Chol specifically delivered to PC-3 cells

**Fig 3:**
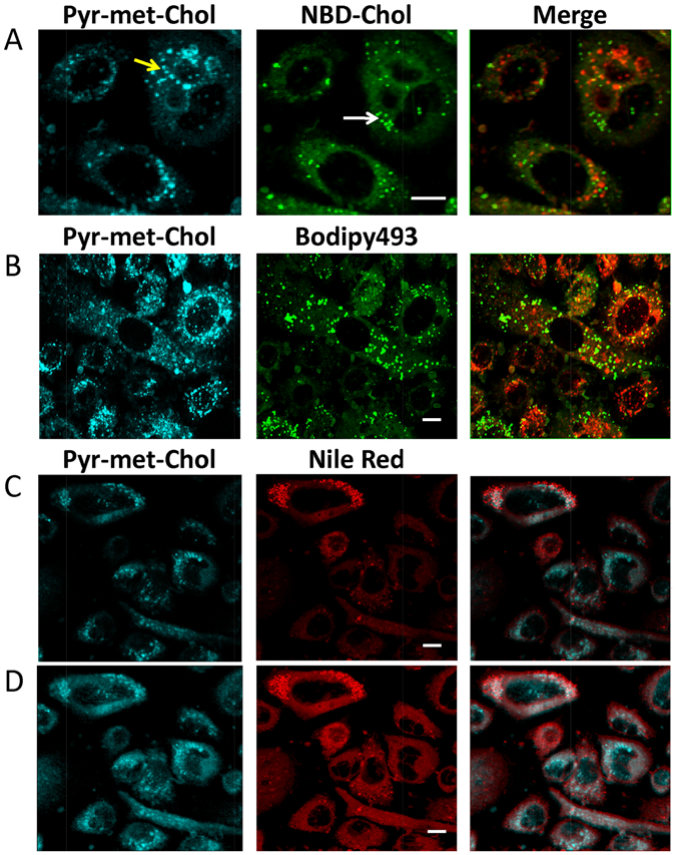
Pyr-met-Chol staining of PC-3 cells. *Panel A*: Double staining of PC-3 cells by Pyr-met-Chol (cyan channel) and NBD-Chol (green channel). PC-3 cells were incubated for 48 h in Ham F12 medium supplemented with 10% fetal calf serum in the presence of 5 μM Pyr-met-Chol and 5 μM NBD-Chol; cells were then fixed and observed by TPE fluorescence microscopy. Arrows indicate examples of the intracellular structures intensely stained by either fluorescent cholesterol derivative. The third column is the merge image, channel superimposition is in red. Scale bar corresponds to 10 μm. *Panel B*: Double staining of PC-3 cells by Pyr-met-Chol (cyan channel) and Bodipy493 (green channel). PC-3 cells were incubated with Pyr-met-Chol as above, then fixed and treated with 1 μg/ml Bodipy493, and observed by TPE microscopy. The third column is the merge image, channel superimposition is in red. Scale bar corresponds to 10 μm. *Panels C and D*: Double staining of PC-3 cells by Pyr-met-Chol (cyan channel) and Nile Red (red channel). PC-3 cells were incubated with Pyr-met-Chol as above, then fixed and treated with 1 μg/ml Nile Red, and observed by TPE microscopy. The third column is the merge image, channel superimposition is in white. Scale bar corresponds to 10 μm. In panel D, the images were obtained from panel C by increasing the gamma factor to 2, which enhances the low and medium intensity red pixels without modification of the brightest ones, thus allowing for a clear vizualization of the diffuse cytoplasmic staining as well as brighter structures (but no more allowing relative quantifications).

We then addressed intracellular location of Pyr-met-Chol delivered through lipoprotein-dependent pathways. For the sake of comparison, we first analyzed the respective cellular fluorescent staining patterns obtained after 48 h incubation with Pyr-met-Chol and NBD-Chol simultaneously added (each at 5 μM) to the serum-containing culture medium. After cell fixation, the co-staining images showed that the fluorescence emissions of Pyr-met-Chol (cyan) and of NBD-Chol (green) were mainly observed in intensely stained intracellular punctate structures (Fig 3A). However, these structures clearly did not colocalize, as demonstrated in the merge image, the channel superimposition being mainly limited to regions corresponding to diffuse cytoplasmic staining by Pyr-met-Chol and NBD-Chol, with the only exception of very few punctate structures apparently overlapping (within the image resolution). When we stained PC-3 cells with Bodipy493, a classical marker of intracellular lipid bodies, we also observed that Pyr-met-Chol fluorescence virtually did not colocalize with the structures stained by Bodipy493 (Fig 3B), confirming NBD-Chol data. Anyway, it could be expected that both cholesterol derivatives can partially colocalize within some intracellular membranes.

These imaging data were then reinforced by biochemical analysis of the capacity of PC-3 cells to esterify the cholesterol derivatives. After a 72 h incubation with these two cholesterol derivatives, separation by thin layer chromatography of the cell lipid extracts showed a large fluorescent spot for NBD-Chol (R_f_ = 0.035) and a small spot for esterified NBD-Chol (R_f_ = 0.162), but only one detectable fluorescent spot for Pyr-met-Chol (R_f_ = 0.129), indicating that ACAT was not able to esterify Pyr-met-Chol. In addition, when PC-3 cells were incubated with TMP-153, an ACAT inhibitor, we noticed a decrease in number and intensity of the punctate structures stained by NBD-Chol, while the structures intensely stained by Pyr-met-Chol displayed a decreased intensity that follows the global cell staining (panels A and B in S3 Fig). Actually, under the same conditions, we observed a marked inhibition of Pyr-met-Chol cellular incorporation by about 55% (p<5%) (panel C in S3 Fig), whereas no effect was detected for NBD-Chol (panel D in S3 Fig). These experiments using TMP-153 also showed that the intracellular esterification of cholesterol regulated the entry of Pyr-met-Chol delivered by lipoprotein-dependent pathway, but not that of NBD-Chol, which was delivered by a non-specific route.

When PC-3 cells were stained with Nile Red, whose red fluorescence emission non-specifically probes membrane polar lipids [38], there was a partial but fair colocalization with Pyr-met-Chol staining (Fig 3C), indicating that Pyr-met-Chol inserts into various intracellular membranes. Indeed, by altering the image contrast, it could be observed that, besides the intensely stained intracellular structures, Pyr-met-Chol staining also displayed a diffuse cytoplasmic staining (in agreement with the previous observations of non-fixed cells) that colocalized with Nile Red (Fig 3D).

For the sake of comparison, we also observed PC-3 cells stained with filipin, the classical marker of unesterified cell cholesterol. The staining pattern showed a well-labelled plasma membrane and some intracellular irregular structures (S4 Fig). Comparison with Pyr-met-Chol staining reflects the inherent difference between the two fluorophores, that is filipin stains all the cellular non-esterified cholesterol pools while Pyr-met-Chol can only trace for the exogenous cholesterol delivered through lipoproteins pathways.

### Intracellular distribution in PC-3 cells of Pyr-met-Chol specifically delivered by LDL or HDL

**Fig 4:**
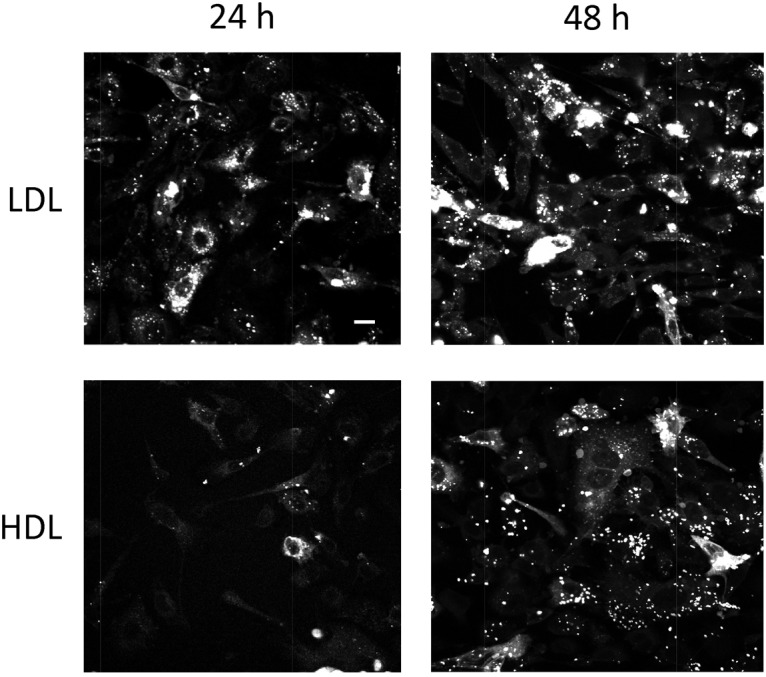
PC-3 cells staining by Pyr-met-Chol-labelled purified HDL or LDL. TPE microscopy imaging of fixed PC-3 cells was performed after a 24 h (left panels) or 48 h (right panels) incubation in the presence of 0.1 mg/ml of Pyr-met-Chol-labelled purified LDL (upper panels) or Pyr-met-Chol-labelled purified HDL (lower panels). TPE microscopy acquisitions were done with the same gain settings for the 24 h and 48 h incubation conditions, but they were lower for LDL than for HDL. Scale bar corresponds to 10 μm.

Since specific lipoprotein-dependent pathways appear to be involved in Pyr-met-Chol cellular uptake, we further focused on the comparison of intracellular compartments stained by Pyr-met-Chol when delivered to PC-3 cells by either purified human LDL or HDL. TPE microscopy observations showed that Pyr-met-Chol staining of the punctate structures was more important for LDL than for HDL, and clearly increased between 24 h and 48 h for HDL incubation, while roughly constant with LDL (Fig 4). This is in agreement with the cellular incorporation kinetics displayed in Fig 2B, and allows to conclude that the intense staining of these intracellular structures is roughly proportional to the global cell incorporation (without major bias due to any quenching or enhancement phenomenon).

**Fig 5:**
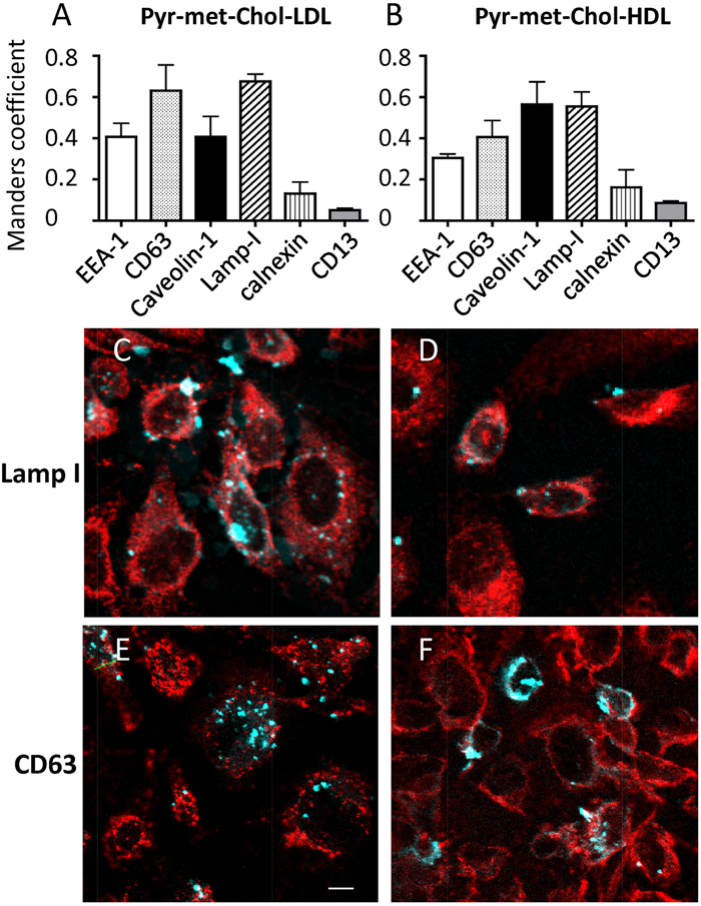
Colocalization analysis between Pyr-met-Chol and protein markers of intracellular membrane compartments in PC-3 cells. TPE microscopy imaging of fixed PC-3 cells was performed after a 48 h incubation with 0.1 mg/ml of Pyr-met-Chol-labelled purified LDL (left panels) or Pyr-met-Chol-labelled purified HDL (right panels), followed by permeabilization with Triton X-100 and immunostaining using specific antibodies against either EEA-1 (early endosomes), CD63 (prostasome-precursor vesicles), caveolin-1 (caveolae), Lamp-1 (late endosomes), calnexin (endoplasmic reticulum) or CD13 (plasma membrane). *Panels A and B*: Manders coefficient were calculated as indicators of the proportion of Pyr-met-Chol signal in the intensely stained structures that colocalized with the signal of each of the antibodies (secondary antibodies labelled by Cy3, except Alexa-546 for caveolin-1 detection). *Panels C and D*: Merge images of the colocalization of Pyr-met-Chol (cyan channel) with Lamp-1 (red channel); superimposition is in white. *Panels E and F*: Merge images of the colocalization of Pyr-met-Chol (cyan channel) with CD63 (red channel); superimposition is in white. Scale bar corresponds to 10 μm.

We then determined the intracellular distribution of the Pyr-met-Chol-stained structures by analyzing their colocalization with various specific markers of intracellular membrane compartments (Fig 5). After 48 h of incubation with either Pyr-met-Chol-labelled LDL or HDL, the stained structures were mainly associated with the late endosome marker Lamp-1 and with the prostasome-precursor intracellular vesicles marker CD63 (Fig 5A and 5B), as examplified by the merge images (Fig 5C and 5F; see the corresponding monochannel images in panels A-D in S5 Fig), with no clear difference for the levels of partial colocalization observed with HDL and LDL incubations. Partial colocalization was also observed with the caveolae marker caveolin-1, but also without clear difference between HDL and LDL incubations. In addition, with both LDL and HDL, Pyr-met-Chol-stained structures colocalized to a lesser extent with the early endosome marker EEA-1, and were virtually not associated with the endoplasmic reticulum marker calnexin, or with the plasma membrane marker CD13.

### Pyr-met-Chol excimer to monomer fluorescence ratio as a specific tool to localize and follow ordered membranes

**Fig 6:**
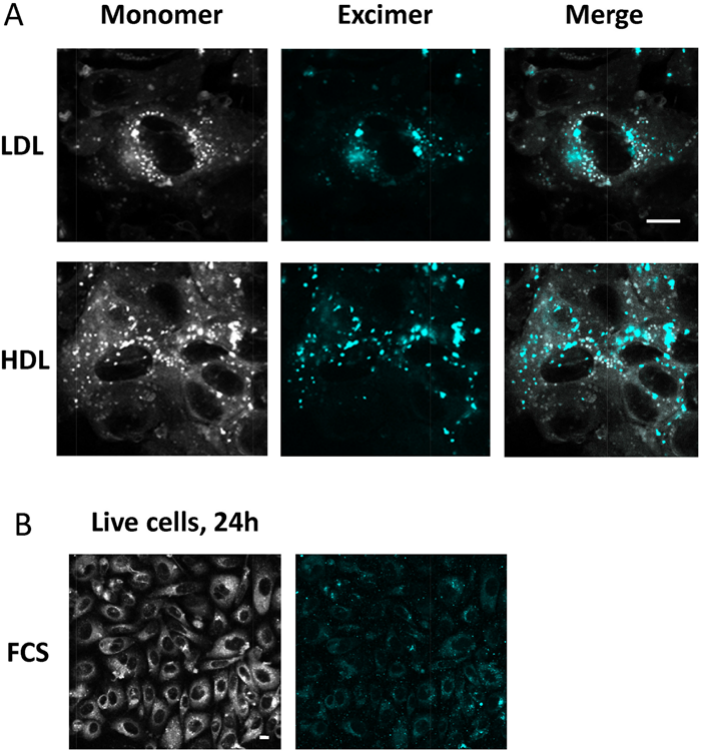
Simultaneous staining of PC-3 cells by Pyr-met-Chol monomers and excimers. *Panel A*: TPE microscopy imaging of fixed PC-3 cells was performed after a 48 h incubation with 0.1 mg/ml of Pyr-met-Chol-labelled purified LDL (upper panels) or Pyr-met-Chol-labelled purified HDL (lower panels), and the fluorescence emission was simultaneously observed for pyrene monomers (white channel) and excimers (cyan channel). Acquisition gains were much higher for images obtained for HDL incubation than for LDL. Scale bar corresponds to 10 μm. *Panel B*: TPE microscopy imaging of living, non-fixed PC-3 cells was performed after a 24 h incubation of Pyr-met-Chol in the presence of 10% fetal calf serum, and the fluorescence emission was simultaneously observed for pyrene monomers (white channel) and excimers (cyan channel). Scale bar corresponds to 10 μm.

Thanks to its pyrene moiety, Pyr-met-Chol has the advantage to form excimers when inserted in raft-like ordered lipid membranes [13], displaying fluorescence emission at higher wavelengths than monomers (420–580 nm vs 360–410 nm). After PC-3 cells were incubated for 48 h with either Pyr-met-Chol-labelled LDL or HDL, the fluorescence emission of Pyr-met-Chol was recorded in both the monomer and excimer channels of the TPE microscope. We observed that, for both LDL and HDL-mediated delivery, the major part of the intracellular structures intensely stained by the Pyr-met-Chol monomers were most intensely stained by Pyr-met-Chol excimers (Fig 6A). An excimer/monomer ratio I_E_/I_m_ could be calculated from the measured fluorescence intensities in the doubly intensely stained structures, considered as regions of interest. This ratio had a similar value for either route delivering Pyr-met-Chol to the cells, LDL (I_E_/I_m_ = 0.62) or HDL (0.65). In both cases, these data show that Pyr-met-Chol excimers can evidence intracellular membrane domains that provide to the dye a specific local environment characterized by an ordered structural organization, and that these membrane compartments were observed to be among those that contain the highest amounts of Pyr-met-Chol incorporated in the cells. In the other hand, the intracellular structures with lower fluorescence emission in the monomer channel, including the diffuse cytoplasmic staining, were not at all apparent in the excimer channel (Fig 6A). These observations that Pyr-met-Chol, delivered via LDL and HDL pathways, preferentially associated to ordered, cholesterol-rich, intracellular membranes, thus indicated a similar distribution to that occuring with membrane cholesterol.

We then observed living, non-fixed PC-3 cells in both monomer and excimer channels over a 24 h Pyr-met-Chol incubation in the presence of serum. After 5 h, both Pyr-met-Chol monomer and excimer stainings were visible, but rather faint (not shown), in agreement with the cellular incorporation kinetics presented in Fig 2A. After 24 h, excimer staining mostly corresponded to the intracellular punctate structures stained by the monomers (Fig 6B). The excimer/monomer ratio I_E_/I_m_ was evaluated to 0.78 under these conditions. These observations on living cells showed that cell fixation did not sensibly alter the staining pattern exhibited by Pyr-met-Chol.

### Functional relevance of the Pyr-met-Chol stained membranes

**Fig 7:**
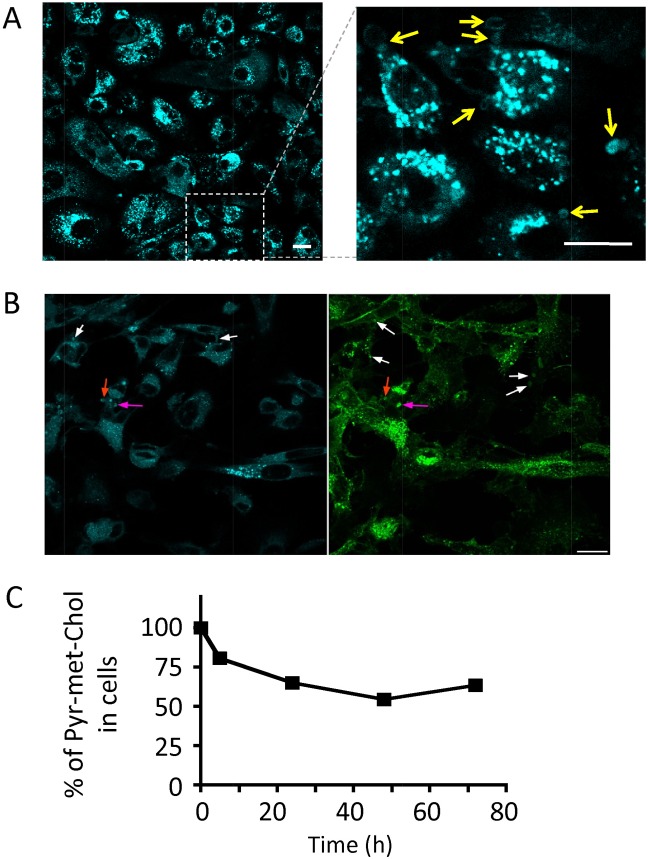
Staining of pericellular membrane structures and Pyr-met-Chol efflux. *Panel A*: Pyr-met-Chol staining of pericellular structures. TPE microscopy imaging of fixed PC-3 cells was performed after a 48 h incubation of 5 μM Pyr-met-Chol in the presence of 10% fetal calf serum. Scale bar corresponds to 10 μm. The right image is a magnification of the left one, and the arrows point some of the pericellular structures. *Panel B*: Fluorescent labelling of transfected PC-3 cells expressing the fusion protein EGFP-SR-BI. TPE microscopy imaging of fixed PC-3/EGFP-SR-BI cells was performed as in panel A (48 h incubation of 5 μM Pyr-met-Chol in the presence of 10% fetal calf serum); cyan channel for Pyr-met-Chol and green channel for EGFP-SR-BI; the white arrows point some of the singly labelled pericellular structures, and the red and pink arrows point two doubly labelled pericellular structures. *Panel C*: Kinetics of Pyr-met-Chol efflux from PC-3 cells. PC-3 cells were incubated for 48 h with 5 μM Pyr-met-Chol in the presence of 10% fetal calf serum, then washed and further cultured in the absence of Pyr-met-Chol for various periods. Remaining Pyr-met-Chol content was measured by fluorometry on cells lysed in the presence of 0.5% SDS. When non-apparent, error bars are included within the symbols.

Pyr-met-Chol staining (after a 48 h incubation) of PC-3 cells gave the opportunity to further study their ordered membrane pools. In particular, besides the intensely stained punctate intracellular structures described above, we also observed rather large and irregularly shaped, often hollow, structures at the periphery of some of the cells (Fig 7A). These structures were present in spite of cell washing, indicating that they were likely not free but somehow attached to the cells. These “pericellular structures” were also observed after Nile Red staining (Fig 3C), which showed their membranous nature. They were similarly observed on living, non-fixed cells (not shown). However, they were no longer apparent after permeabilizing detergent treatment, precluding any immunofluorescence study of membrane markers associated with these structures. In addition, when Pyr-met-Chol was delivered from either purified LDL or HDL, these pericellular structures were approximately similarly present (not shown). By analyzing Pyr-met-Chol cell staining of these structures in both monomer and excimer channels of the TPE microscope, the excimer/monomer ratio I_E_/I_m_ was evaluated to 0.37 after LDL-mediated Pyr-met-Chol delivery and to 0.20 after HDL-mediated Pyr-met-Chol delivery. Finally, since it has been reported that the microvillar extensions present on various cell lines are the preferential location of SR-BI [39], we engineered the PC-3/EGFP-SR-BI cells to address the possible relationships between SR-BI and the pericellular structures. It is noteworthy that these pericellular structures were labelled in these cells expressing the fusion fluorescent protein EGFP-SR-BI (Fig 7B).

We then analyzed Pyr-met-Chol efflux out of PC-3 cells prelabelled by a 48 h Pyr-met-Chol incubation in the presence of serum, and washed before further culture in the absence of Pyr-met-Chol. We prepared a membrane fraction by ultracentrifugation of the culture medium collected after 96 h, as previously described [40], and we recorded the fluorescence emission spectrum of these membranes. This showed that a noticeable fraction of the secreted Pyr-met-Chol emitted in the excimer wavelengths (I_480_/I_400_ = 0.24), indicating that at least a fraction of the dye was present in secreted ordered membranes. Finally, we measured kinetics of Pyr-met-Chol efflux over 72 h from the cells prelabelled under the same conditions, and this showed that a major part (roughly half) of Pyr-met-Chol incorporated into the PC-3 cells was not retained but was secreted by the cells, with a relatively slow rate (half-time of 5–6 hours, Fig 7C).

## Discussion

In contrast to NBD-Chol, Pyr-met-Chol is shown here to use specific pathways, involving SR-BI and LDL-R, to incorporate two different cultured cell types, i.e. biliary micelles for Caco-2 cells and labelled lipoproteins for PC-3 cells. This evidence is pivotal to validate the physiological relevance of the observed characteristics of the intracellular trafficking of the considered fluorescent sterol. In particular, after a 48h incubation with PC-3 cells, Pyr-met-Chol is observed to preferentially localize in cholesterol-rich ordered intracellular membranes after trafficking downwards HDL as well as LDL receptors.

### Distinct cell incorporation fates for NBD-Chol and Pyr-met-Chol from biliary micelles

Caco-2 cell line displays an enterocytic differentiation phenotype which makes it a valuable model for studying intestinal absorption processes, including cholesterol transport. For lipid absorption to be realized at the level of the apical brush border, bile salt-based mixed micelles are required to solubilize the dietary lipids and conveniently “present” them to the apical cell transporters in charge of them [41,42]. The use of Caco-2 cells thus gave the opportunity to test the cell uptake of fluorescent cholesterol derivatives using a “simple” delivery route based on bile salt micelles as vectors. We used taurocholate to form model micelles of simplified composition, but aimed at mimicking at best the physiological processes for delivering the fluorescent cholesterol derivatives [43]. However, taurocholate concentration is determinant for the nature and chemico-physical properties of the formed hydrophobic donor structures. Indeed, 5 mM taurocholate (although somehow lower than the cmc of the pure detergent in water) is enough to form mixed micelles in the presence of hydrophobic environment [44,45], while at 2 mM it can be only expected to form so-called “sub-micellar aggregates” [46,47]. Mixed micelles [48] are well-recognized to increase solubility of polycyclic hydrophobic molecules, including sterols, providing an efficient means for emulsification and vectorization [49,50], whereas sub-micellar aggregates can provide only limited solubilization capacities for hydrophobic compounds [47,49]. Data presented in Fig 1A and 1B thus give the indication that Pyr-met-Chol takes much larger benefit of being solubilized in mixed micelles than NBD-Chol in terms of incorporation into Caco-2 cells. In addition, the clear inhibition of Pyr-met-Chol cell incorporation by BLT-1 shows that SR-BI mediates, at least partly, its apical handling, as previously reported for cholesterol after its solubilization in taurocholate-based micelles [41]. In agreement, the fusion protein EGFP-SR-BI, expressed in transfected Caco-2/EGFP-SR-BI cells, was observed to be mainly localized in the apical pole of the cells, even in the absence of any biliary micelles (S6 Fig). In contrast to Pyr-met-Chol, the absence of BLT-1 effect on NBD-Chol cell incorporation, along with the only slight influence of the chemico-physical state of the donor solution that indicated a much lower dependence on micelle-mediated solubilization, is consistent with a purely passive mechanism for cell membrane permeation of this amphiphilic molecule.

Once present in the Caco-2 cells, the two fluorescent cholesterol derivatives also exhibit clearly distinct behaviors. For NBD-Chol, and in contrast to Pyr-met-Chol, colocalization with adipophilin and absence of kinetic saturation of the incorporated amount at 24 h (Fig 1G and 1H) are consistent with its efficient intracellular esterification, allowing accumulation in the lipid bodies, as previously reported for various cell types [14,15,16]. The cellular efflux kinetics also showed differences, being more rapid and important for NBD-Chol than for Pyr-met-Chol. However, for both cholesterol probes, the effluxed fraction with respect to the cellular incorporation at 24 h is fairly comparable (10–15% range) with the reported values (about 5%) using radio-labelled cholesterol on Caco-2 cells in monolayer for the same time range [51].

The marked differences in cell staining patterns observed for the two fluorescent cholesterol probes suggest that they follow distinct dynamic membrane pools when they are incorporated by the delivery system made of taurocholate-based micelles. Since another cell incorporation route for lipids and lipophilic compounds is provided by lipoproteins, we took benefit of the recent evidence that Pyr-met-Chol, in contrast to NBD-Chol, tightly associates with LDL and HDL [19]. We thus further compared these two fluorescent derivatives of cholesterol regarding their cell delivery to the PC-3 cell line model that well expresses lipoproteins receptors.

### Lipoprotein receptor-mediated delivery of Pyr-met-Chol to PC-3 cells

When incubated in the absence of serum with PC-3 cells, Pyr-met-Chol is delivered as a free molecule, and it displays a higher total incorporation and a lower diffuse cytoplasmic staining (relative to the intracellular punctate structures) than in the presence of serum (Fig 2A and 2E). These clear-cut differences of interaction with the PC-3 cells characterize the distinction between non-receptor, i.e. without serum, and receptor-mediated, i.e. with lipoprotein-containing serum, influx pathways. In the last case, no Pyr-met-Chol appears to be exchanged with the incubation medium as free molecules (as would happen if the lipoprotein labelling would have been not so stable), and all Pyr-met-Chol is thus sequestered by serum components, that sustain a limiting and specific cellular incorporation, even in the early phases of incubation. Albumin, well-known to bind lipophilic molecules and alter their bioavailability [52], cannot directly be involved in mediating Pyr-met-Chol cell delivery (Fig 2C), but may contribute as an intermediate chelating component that would transiently transfer Pyr-met-Chol to lipoproteins for receptor-mediated cell delivery. In addition, it should be noted that the slower cell incorporation kinetics of Pyr-met-Chol observed when it is directly added to serum (Fig 2C) rather than using labelled purified LDL (Fig 2B) was likely a result of the delay (hour range) necessary for labelling the LDL present in the serum [19].

The involvement of lipoprotein receptors is confirmed by the decrease of Pyr-met-Chol incorporation observed with serum in the presence of the LXR agonist, 25-hydroxycholesterol (panel A in S2 Fig), or the ACAT inhibitor, TMP-153 (panel C in S3 Fig), two compounds known to alter receptor-mediated cholesterol incorporation [53]. Moreover, the higher and faster cellular incorporation from Pyr-met-Chol-labelled purified LDL than from Pyr-met-Chol-labelled purified HDL (Fig 2B) is also indicative of an interaction with the corresponding specific receptors, and this is consistent with the reported overexpression of LDL receptors in the PC-3 cells [21,35]. However, when PC-3 cells are incubated in the presence of total serum, the relative contributions of LDL- and HDL-mediated incorporation routes are expected to be fairly comparable since HDL are present in foetal calf serum in somehow higher (2–3 fold) amounts than LDL [54].

At variance with Pyr-met-Chol, NBD-Chol cellular incorporation is not sensitive to 25-hydroxycholesterol or TMP-153, confirming that NBD-Chol, which virtually does not bind to lipoproteins, is delivered to the cells by a receptor-independent incorporation pathway that is only partially limited by its chelation to albumin (Fig 2D). Thus, in PC-3 as well as in Caco-2 cells, Pyr-met-Chol and NBD-Chol are submitted to receptor-mediated and non-specific cellular incorporation pathways, respectively.

### Intracellular staining by lipoprotein-delivered Pyr-met-Chol

#### Compared intracellular esterification of the two cholesterol derivatives

When Pyr-met-Chol and NBD-Chol are simultaneously delivered to PC-3 cells in the presence of serum, they intensely stain internal structures that are clearly distinct (Fig 3A), evidencing different intracellular routing for these two fluorescent cholesterol derivatives. NBD-Chol well labels lipid bodies (or droplets) [14,15], displaying a dramatically increased fluorescence emission when included as esterified form in the hydrophobic core of these particles. Indeed, we show that NBD-Chol is partly esterified in PC-3 cells, although TMP-153 does not affect NBD-Chol cell incorporation (panels B and D in S3 Fig), which is likely the consequence of the esterification of only a small fraction of the probe, assuming that esterification is necessary for increasing the amount of intracellular stored sterols. In contrast, Pyr-met-Chol labels intracellular structures different from lipid bodies, and is not detectably esterified by ACAT in PC-3 cells. The partial colocalization (15%) of the diffuse Pyr-met-Chol cytoplasmic staining with the marker calnexin (not shown) rules out an impediment of access to ACAT in the endoplasmic reticulum. Otherwise, this absence of intracellular Pyr-met-Chol esterification is consistent with the reported dependence of ACAT activity on the structure of the lateral chain presented by sterols, such as phytosterols [55,56]. Such metabolisation difference between NBD-Chol and Pyr-met-Chol has also been observed for lecithin:cholesterol acyltransferase (LCAT) [19].

#### Compared incorporation routes mediated by LDL and HDL

Since we have recently shown the specific association of Pyr-met-Chol with LDL and HDL [19], we are in position to compare their respective cell incorporation routes. When either Pyr-met-Chol-labelled purified LDL or Pyr-met-Chol-labelled purified HDL were added to PC-3 cells, the staining intensity of intracellular stuctures increases faster and more intensely with LDL than with HDL (Fig 4), in agreement with the cell incorporation kinetics (Fig 2B). However, the low staining signal for short-time incubation (< 24h) does not allow to discriminate between a time shift or an actual qualitative difference between HDL and LDL routes. Anyway, at steady state (> 48h), no clear difference between LDL- and HDL-mediated delivery was observed for Pyr-met-Chol staining, regarding colocalization with various membrane compartment markers or the fluorescence ratio excimer/monomer in the intensely stained structures. Actually, LDL-labelling Pyr-met-Chol, incorporated by the "LDL receptor-mediated clathrin-dependent endocytosis" mechanism [57], and HDL-labelling Pyr-met-Chol, incorporated by the "HDL retro-endocytosis" mechanism [58], finally meet toward a common intracellular compartment, likely related to late endosomes (Lamp-1 positive) and multivesicular bodies (CD63 positive), where Pyr-met-Chol accumulates lately (Fig 5). Thus, there is no indication of durably separated pools of intracellular Pyr-met-Chol either delivered from LDL or HDL, but rather a progressive “mixing” in the various intracellular membranes of these two exogenous fluorescent cholesterol pools of different origins. To our knowledge, such a question has never been directly addressed using a specific fluorescent labelling of the donor LDL vs HDL particles, and this evidences the usefulness of Pyr-met-Chol as a marker of specific lipoprotein-mediated cell delivery.

### Pyr-met-Chol excimer fluorescence emission as a marker of intracellular cholesterol-rich membranes

An interesting hallmark of the pyrene group is its ability to form excimers, displaying a specific fluorescence emission under favorable local conditions. In particular, Pyr-met-Chol can reveal ordered, cholesterol-rich, raft-like microdomains in model membranes [13], with a ratio of excimer to monomer emission in the range of 0.2–1 (depending on the lipid composition), this value being roughly independent from the amount of probe present in the membrane [59]. In our experiments, the excimer emission in the intracellular structures is similar when Pyr-met-Chol is delivered from LDL or HDL, with a ratio excimer to monomer evaluated by double channel TEP microscopy at about 0.6 (Fig 6A), although the amounts of dye incorporated differs by a factor of about 4. This indicates that Pyr-met-Chol excimer emission cannot be due to a simple concentration effect, but reflects the ordered organization of the membrane environment of the dye, likely linked to a local enrichment in cholesterol. This environment felt by the probe downwards lipoprotein uptake is obviously independent of that occuring within the fluorescently labelled lipoproteins that are incubated with the cells [19]. In addition, the majority of intracellular structures that display excimer emission are also highly stained by Pyr-met-Chol monomers, suggesting that Pyr-met-Chol and cholesterol distribute similarly, at least in the cholesterol-rich membranes. Indeed, these Pyr-met-Chol-stained intracellular structures colocalize with markers of late endosomes and of multivesicular bodies, both known for their high cholesterol content [60], and with caveolin-1, well-known for its association with cholesterol. These data validate the usefulness of this cholesterol-derived probe as a convenient tool for addressing the cellular fluxes of cholesterol-rich membranes (without the drawbacks of cell fractionation).

As an illustration, PC-3 cells, which are well-known for their capacity to secrete cholesterol- and sphingomyelin-rich membrane vesicles, called prostasomes [61], harbor pericellular structures that are stained by Pyr-met-Chol monomers and excimers (Fig 7A). These membrane structures could be reminiscent of the blebs (or “aposomes”) reported to occur in various prostate cells [62]. The colocalization in at least a part of these pericellular structures of Pyr-met-Chol and EGFP-SR-BI (Fig 7B), which otherwise labels the plasma membranes as expected for its function as HDL receptor, raises the question of possible functional relationships between them, in particular in the frame of the multifunctional abilities of SR-BI. Finally, incorporated Pyr-met-Chol is not sequestered in the cells, and can follow outward exchanges with the external medium, as shown by its efflux kinetics (Fig 7C) and its presence in extracellular membrane vesicles. Pyr-met-Chol can thus be followed for its cell influxes and effluxes whatever the exchange mechanisms involved, such as bile salt micelles and lipoproteins, respectively, as observed with Caco-2 line, or lipoproteins and membrane vesicles, respectively, as observed with PC-3 line.

### Pyr-met-Chol, a new fluorescent probe to investigate membrane cholesterol trafficking in target cells downwards specific delivery

Few years ago, Pyr-met-Chol has been synthesized as a new pyrene-labelled cholesterol derivative [13], differing from a previously synthesized pyrene-based cholesterol derivative using a carboxyl group for grafting the fluorophore, which had the drawback of giving too a hydrophilic molecule for reliably mimicking cholesterol membrane interaction [63,64]. Indeed, Pyr-met-Chol compares favorably with other fluorescent lipid probes already used as markers of lipoprotein delivery since, typically, DiI and DiO are widely used as fluorescent membrane probes delivered to target cells by labelled lipoproteins [65,66], but the charged structure of the carbocyanine moiety makes it sensitive to membrane potential, thereby influencing its intracellular distribution. Moreover, the length and saturation of the alkyl chains of DiI/DiO are determining for targeting the membrane compartments where they insert, which can be influenced by the presence of microdomains [67]. Among the very few fluorescent cholesterol derivatives studied on cultured cells [8], generally using non-specific routes for cell delivery, only HDL-mediated cell delivery of DHE [9] and of Bodipy-Chol [10] have been tested. With respect to Bodipy-Chol, Pyr-met-Chol shares some interesting and important biological properties, such as good photostability and preferential partitioning in cholesterol-rich membranes, but however they essentially differ by their ability to be esterified in cells: Bodipy-Chol is esterified, but in a more or less lower level than (radioactive) cholesterol depending on the incubation conditions of the cultured cells (but using non-specific routes for cellular delivery) [11], whereas Pyr-met-Chol is not esterified, which could have some consequences on their intracellular trafficking and distribution. Indeed, Pyr-met-Chol can be considered as a fair probe of non-esterified cholesterol, and thus a good reporter in the cells of interest of the intra-membrane cholesterol of exogenous origin, without any interference with esterification metabolism, including various cholesterol pools handled by the two ACAT isoforms and their possible regulations. Otherwise, pyrene-labelled phospholipids [68] or cholesterol esters delivered by LDL [69] have been used to investigate lipid metabolism, but since these probes are processed by enzymatic activities, they cannot reliably trace membrane compartments. It appears finally that Pyr-met-Chol and Bodipy-Chol exhibit distinct but complementary cellular properties, and can hence be considered as aimed at probing different aspects of cholesterol cell handling.

As a whole, Pyr-met-Chol presents many advantages: (i) a long-term metabolic stability and a strong resistance to photodegradation, in contrast to DHE, cholestatrienol and dansyl-cholesterol; (ii) the chemico-physical properties of Pyr-met-Chol for insertion in the membrane lipid phase, which allows to trace the cholesterol molecules, in particular regarding partitioning into cholesterol-rich ordered domains; (iii) the ability of Pyr-met-Chol to follow specific cell delivery routes, which validates the physiological relevance of its observed intracellular trafficking; (iv) the possible simultaneous observations for Pyr-met-Chol of two fluorescence emission wavelengths (corresponding to monomers and excimers) that report on its molecular environment, which allows a quantitative follow-up of a given membrane compartment corresponding to a measured emission excimer/monomer ratio.

Actually, Pyr-met-Chol can stain various internal membrane compartments, more or less enriched in cholesterol, including endoplasmic reticulum where Pyr-met-Chol is even more prominent (~15%) than cholesterol (representing less than 2% [2]), perhaps as a consequence of not being esterified by ACAT. It should be noted that the very weak Pyr-met-Chol staining of plasma membrane is in agreement with cell labelling by DHE, that is much less intense than filipin at the plasma membrane level [70]. This could be interpreted by the fact that the cholesterol pool in plasma membrane is mainly provided from endogenous biosynthesis, feeding this important membrane with intact, non-oxidized cholesterol, at variance with exogenous cholesterol possibly delivered by oxidized lipoproteins. Although it remains to be determined whether Pyr-met-Chol is submitted to "regular" interactions with the various proteins involved in the intracellular cholesterol trafficking, Pyr-met-Chol finally appears to much more reliably trace the physiological traffic pathways of exogenous unesterified cholesterol than NBD-Chol does.

## Conclusion

As a conclusion, Pyr-met-Chol is a reliable fluorescent exogenous sterol that can probe bile salt micelle-mediated as well as lipoprotein-mediated specific cellular delivery, evidencing in both cases the involvement of the multifunction scavenger receptor SR-BI. It is also a probe that gives the opportunity to delineate the respective cellular pathways downwards the HDL and LDL receptors. Since this probe allows to conveniently observe the intracellular pathways involving cholesterol-rich membranes, it could be considered as a fair tracer of exogenous unesterified cholesterol (and other sterols). This approach could hence be applied to any other cell models that have to deal with important cholesterol fluxes with the extracellular medium (e.g. hepatocytes, neurons…).

## Supporting Information

S1 Fig Caco-2 cells costaining either by NBD-Chol and Pyr-met-Chol (panel A) or by NBD-Chol and ADRP immunofluorescence (panel B). *Panel A*: Merge image corresponding to the monochannel images presented in Fig 1E and 1F: green: NBD-Chol localization; blue: Pyr-met-Chol localization. *Panel B*: Caco-2 cells, cultured on glass slides until differentiation, were washed and incubated for 2 h in the presence of biliary micelles containing 5 mM taurocholate and 5 μM NBD-Chol, then fixed and permeabilized, and successively treated by anti-ADRP Ab and by Alexa546-labelled secondary Ab. The cells were observed by bichannel confocal fluorescence microscopy: red, Alexa546 emission; green, NBD emission (yellow signs colocalization). Scale bar correponds to 10 μm.
(TIF)

S2 Fig Effect of 25-hydroxycholesterol and BLT-1 on Pyr-met-Chol incorporation in PC-3 cells. *Panels A and B*: Effect of 25-hydroxycholesterol. PC-3 cells were incubated for 48 h in culture medium supplemented with 10% fetal calf serum in the presence of 5 μM Pyr-met-Chol (A) or 5 μM NBD-Chol (B), in the absence or presence of 10 μM 25-hydroxycholesterol (NT, non-treated control cells). Pyr-met-Chol and NBD-Chol cellular fluorescence emissions were quantified as in Fig 2. p<5% (*) indicates a statistically significant difference. *Panels C and D*: Effect of BLT-1. PC-3 cells were incubated for 48 h in culture medium supplemented with 0,1 mg/ml of Pyr-met-Chol-labelled purified HDL (C) or LDL (D), in the absence or presence of 10 μM BLT-1 (NT, non-treated control cells). Pyr-met-Chol cellular fluorescence emissions was quantified as in Fig 2. p<5% (*) indicates a statistically significant difference.
(TIF)

S3 Fig Effect of the inhibition of cholesterol esterification on Pyr-met-Chol and NBD-Chol incorporation in PC-3 cells. PC-3 cells were incubated for 72 h in culture medium supplemented with 10% fetal calf serum in the presence of 5 μM Pyr-met-Chol (panels A and C) or of 5 μM NBD-Chol (panels B and D), in the absence or presence of 1 μM TMP-153 (NT, non-treated control cells). *Panels A and B*: TPE microscopy imaging was performed as in Fig 3A. Scale bar corresponds to 10 μm. *Panels C and D*: Pyr-met-Chol and NBD-Chol cellular fluorescence emissions were quantified as in Fig 2. p<5% (*) indicates a statistically significant difference.
(TIF)

S4 Fig Staining of PC-3 cells by filipin. PC-3 cells were incubated for 48 h in culture medium supplemented with 10% fetal calf serum. Cells were fixed and treated with 70 μM filipin for 30 minutes at room temperature, and then observed by TPE fluorescence microscopy. Scale bar corresponds to 10 μm.
(TIF)

S5 Fig Monochannel images corresponding to the merge images presented in Fig 5C and 5F. Red channel reports on Cy3 fluorescence emission; cyan channel reports on Pyr-met-Chol fluorescence emission. *Panels A and B*: Lamp-1 detection by Cy3-labelled Abs; *Panels C and D*: CD63 detection by Cy3-labelled Abs; *Panels A and C*: PC-3 cells incubation with Pyr-met-Chol-labelled purified LDL; *Panels B and D*: PC-3 cells incubation with Pyr-met-Chol-labelled purified HDL.
(TIF)

S6 Fig Localization of the fusion protein EGFP-SR-BI expressed in transfected cells Caco-2/EGFP-SR-BI. The transfected Caco-2/EGFP-SR-BI cells were seeded onto glass slides and cultured for 3 days, then induced by 1 μg/ml doxycycline for 1 day, then fixed and observed by TPE fluorescence microscopy. The main image (« XY plane ») is obtained by a Z-cut plane of the cellular monolayer; the rightest image is the YZ plane obtained by a X cut along the vertical white dotted line (« X cut »); the lowest image is the XZ plane obtained by a Y cut along the horizontal white dotted line (« Y cut »); the glass slide level corresponds to the origin of the Z axis. Arrows point the apical side of the cells. Scale bar corresponds to 10 μm.
(TIF)
