# Supplementary figures and images for: Specific Cellular Incorporation of a Pyrene-Labelled Cholesterol: Lipoprotein-Mediated Delivery toward Ordered Intracellular Membranes
Source: PLoS One. 2015 Apr 15;10(4):e0121563. doi: 10.1371/journal.pone.0121563 (PMC4398402; doi:10.1371/journal.pone.0121563)

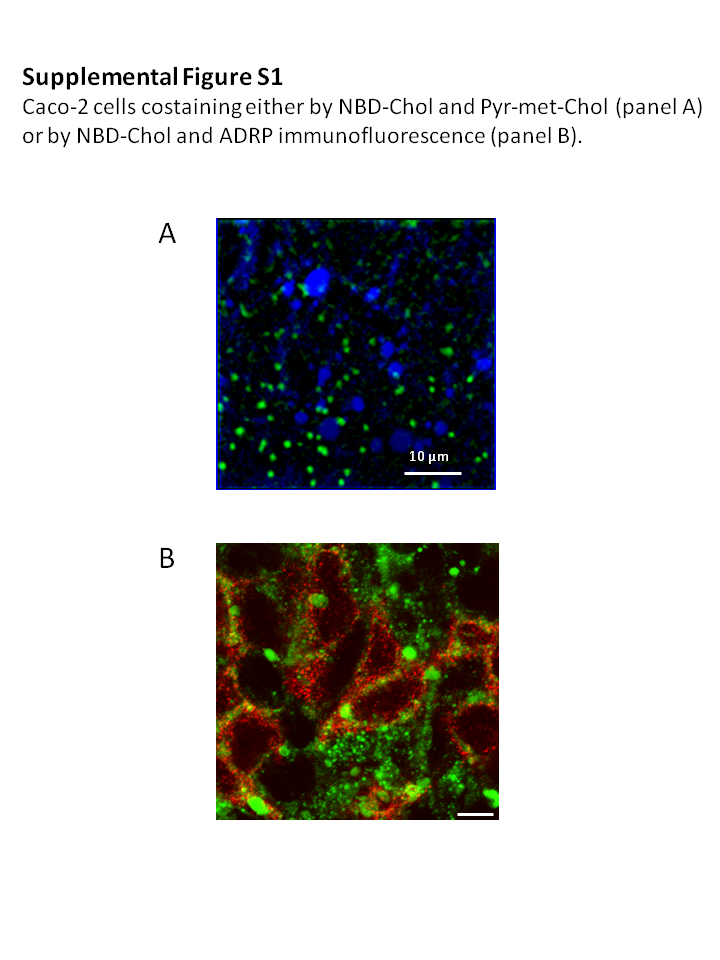

Supplement: S1 Fig — Panel A: Merge image corresponding to the monochannel images presented in Fig 1E and 1F: green: NBD-Chol localization; blue: Pyr-met-Chol localization. Panel B: Caco-2 cells, cultured on glass slides until differentiation, were washed and incubated for 2 h in the presence of biliary micelles containing 5 mM taurocholate and 5 μM NBD-Chol, then fixed and permeabilized, and successively treated by anti-ADRP Ab and by Alexa546-labelled secondary Ab. The cells were observed by bichannel confocal fluorescence microscopy: red, Alexa546 emission; green, NBD emission (yellow signs colocalization). Scale bar correponds to 10 μm. (TIF) [file pone.0121563.s001.tif]

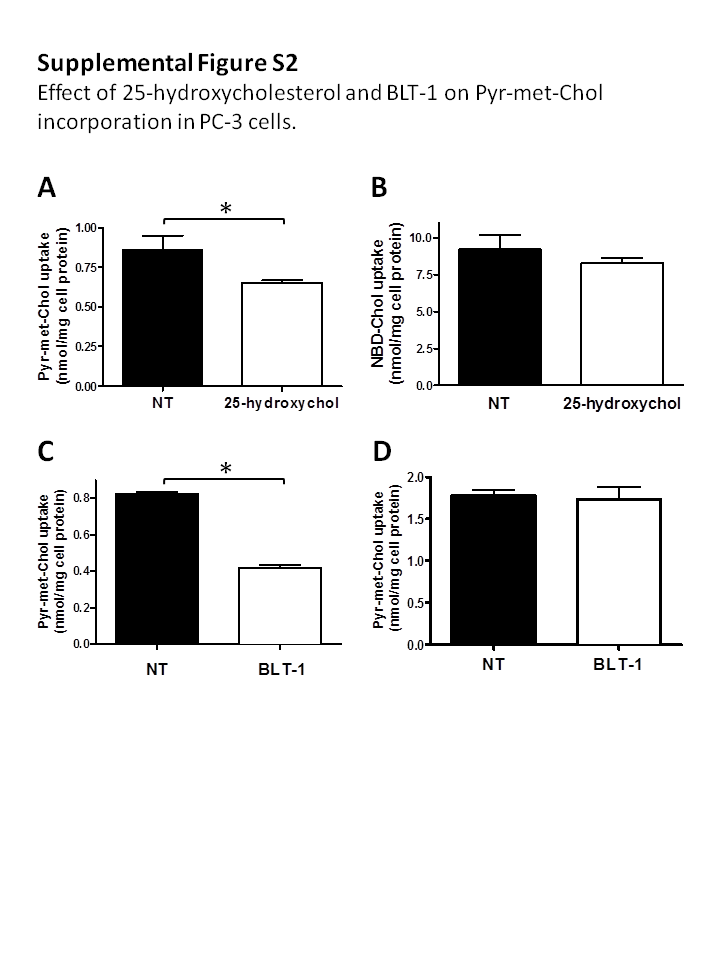

Supplement: S2 Fig — Panels A and B: Effect of 25-hydroxycholesterol. PC-3 cells were incubated for 48 h in culture medium supplemented with 10% fetal calf serum in the presence of 5 μM Pyr-met-Chol (A) or 5 μM NBD-Chol (B), in the absence or presence of 10 μM 25-hydroxycholesterol (NT, non-treated control cells). Pyr-met-Chol and NBD-Chol cellular fluorescence emissions were quantified as in Fig 2. p<5% (*) indicates a statistically significant difference. Panels C and D: Effect of BLT-1. PC-3 cells were incubated for 48 h in culture medium supplemented with 0,1 mg/ml of Pyr-met-Chol-labelled purified HDL (C) or LDL (D), in the absence or presence of 10 μM BLT-1 (NT, non-treated control cells). Pyr-met-Chol cellular fluorescence emissions was quantified as in Fig 2. p<5% (*) indicates a statistically significant difference. (TIF) [file pone.0121563.s002.tif]

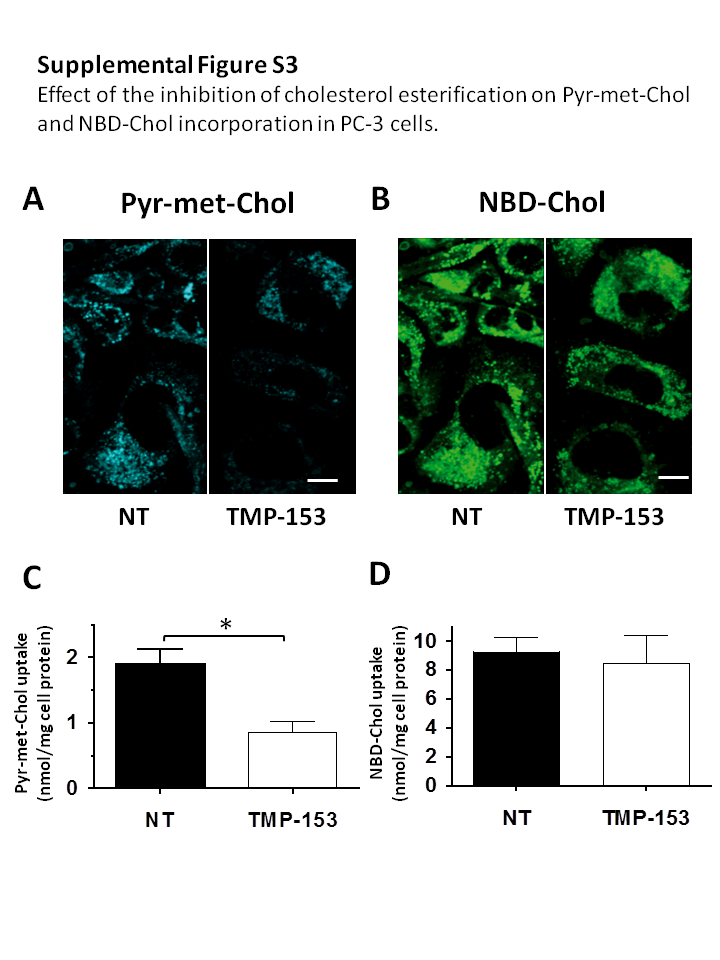

Supplement: S3 Fig — PC-3 cells were incubated for 72 h in culture medium supplemented with 10% fetal calf serum in the presence of 5 μM Pyr-met-Chol (panels A and C) or of 5 μM NBD-Chol (panels B and D), in the absence or presence of 1 μM TMP-153 (NT, non-treated control cells). Panels A and B: TPE microscopy imaging was performed as in Fig 3A. Scale bar corresponds to 10 μm. Panels C and D: Pyr-met-Chol and NBD-Chol cellular fluorescence emissions were quantified as in Fig 2. p<5% (*) indicates a statistically significant difference. (TIF) [file pone.0121563.s003.tif]

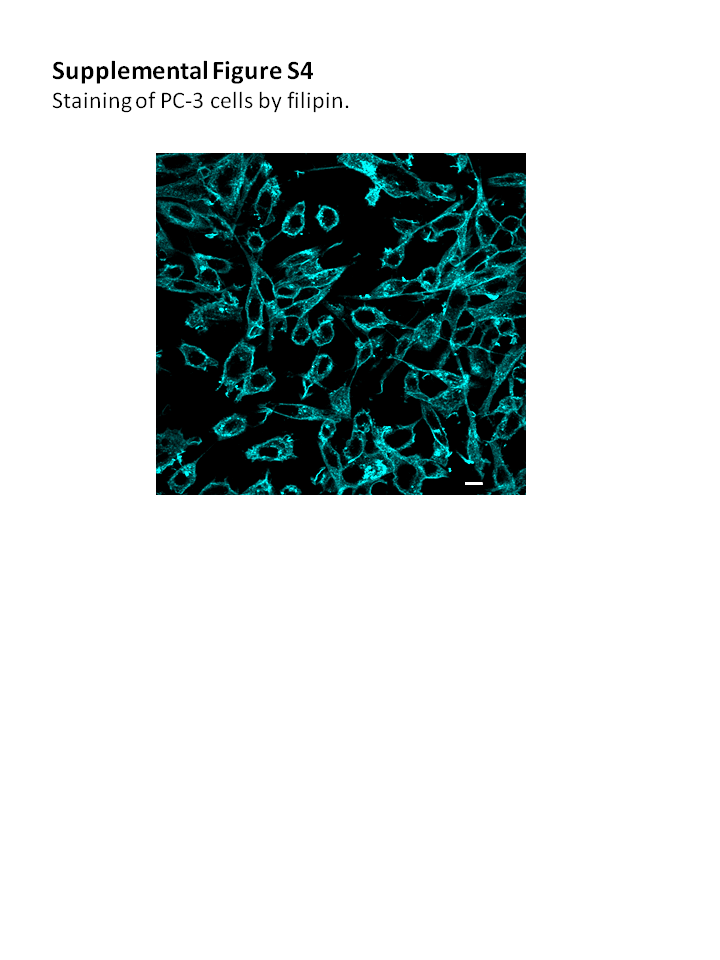

Supplement: S4 Fig — PC-3 cells were incubated for 48 h in culture medium supplemented with 10% fetal calf serum. Cells were fixed and treated with 70 μM filipin for 30 minutes at room temperature, and then observed by TPE fluorescence microscopy. Scale bar corresponds to 10 μm. (TIF) [file pone.0121563.s004.tif]

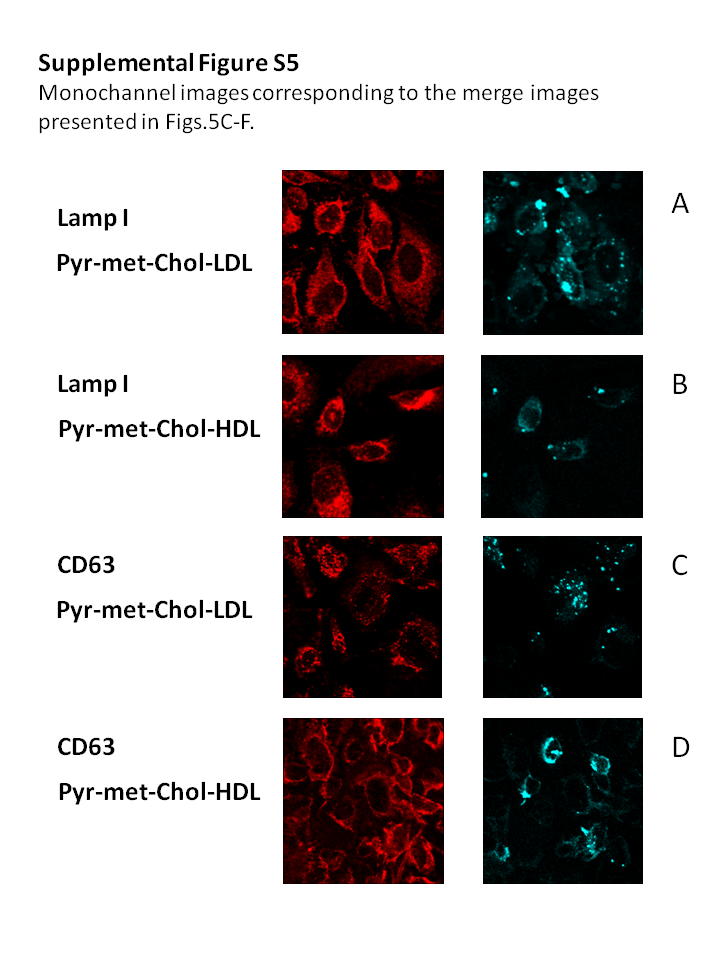

Supplement: S5 Fig — Red channel reports on Cy3 fluorescence emission; cyan channel reports on Pyr-met-Chol fluorescence emission. Panels A and B: Lamp-1 detection by Cy3-labelled Abs; Panels C and D: CD63 detection by Cy3-labelled Abs; Panels A and C: PC-3 cells incubation with Pyr-met-Chol-labelled purified LDL; Panels B and D: PC-3 cells incubation with Pyr-met-Chol-labelled purified HDL. (TIF) [file pone.0121563.s005.tif]

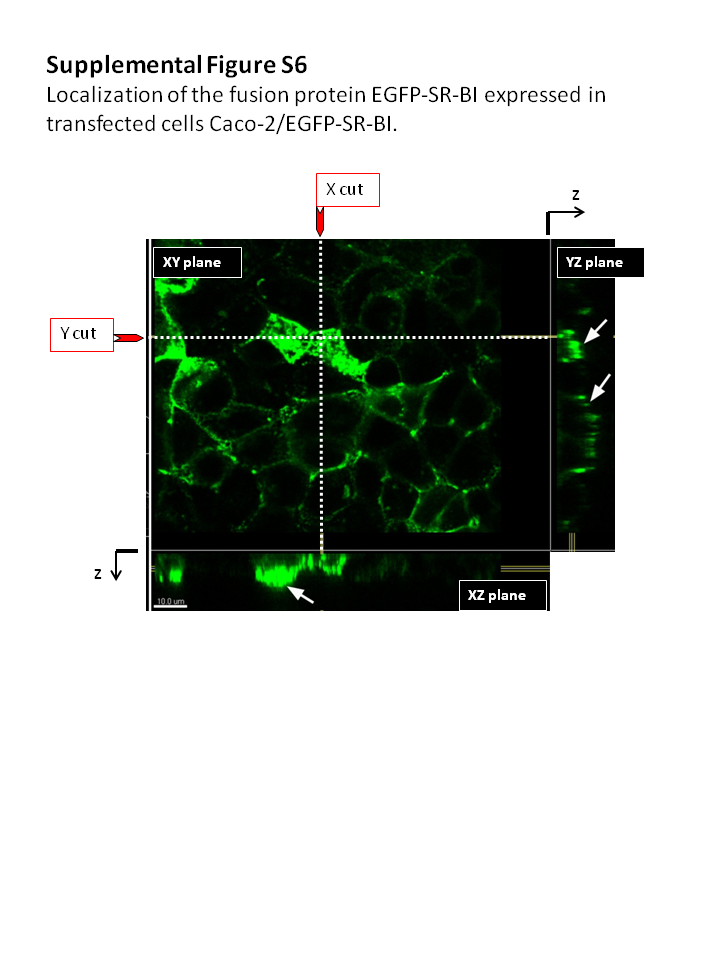

Supplement: S6 Fig — The transfected Caco-2/EGFP-SR-BI cells were seeded onto glass slides and cultured for 3 days, then induced by 1 μg/ml doxycycline for 1 day, then fixed and observed by TPE fluorescence microscopy. The main image (« XY plane ») is obtained by a Z-cut plane of the cellular monolayer; the rightest image is the YZ plane obtained by a X cut along the vertical white dotted line (« X cut »); the lowest image is the XZ plane obtained by a Y cut along the horizontal white dotted line (« Y cut »); the glass slide level corresponds to the origin of the Z axis. Arrows point the apical side of the cells. Scale bar corresponds to 10 μm. (TIF) [file pone.0121563.s006.tif]
